# Association between health‐related quality of life and nonadherence to antihypertensive medication

**DOI:** 10.1002/nop2.1599

**Published:** 2023-01-04

**Authors:** Ka Young Kim

**Affiliations:** ^1^ College of Nursing Gachon University Incheon Korea

**Keywords:** antihypertensive, health‐related quality of life, HRQoL, medication adherence, medication nonadherence

## Abstract

**Aim:**

We aimed to examine the association between nonadherence to antihypertensive medications and Health‐related quality of life.

**Design:**

A cross‐sectional survey was undertaken.

**Methods:**

This study was conducted using the data from the Korea National Health and Nutrition Examination Survey. A total of 6493 participants who diagnosed with hypertension, affected by hypertension at the time of survey, and took daily antihypertensive medications or never took this medication were included. Multiple logistic regression analysis was performed to determine the factors that influenced the patients' nonadherence to antihypertensive medications according to sex.

**Results:**

Our results showed that anxiety/depression was positively associated with antihypertensive medication nonadherence, regardless of sex. According to sex, mobility was negatively associated with antihypertensive medication nonadherence in women. In men, living together was negatively related to antihypertensive medication nonadherence. This study showed the factors associated with antihypertensive medication nonadherence according to sex. HRQoL was associated with antihypertensive medication nonadherence.

## 1 INTRODUCTION

Hypertension was reported as the chief or contributing cause of over half a million deaths in the United States in 2019 (CDC, 2021). Furthermore, hypertension is the leading cause and risk factor for chronic diseases, including stroke, cardiovascular diseases, and renal disease, worldwide; however, it is known to be a preventable and manageable factor (CDC, 2021; WHO, 2021).

Hypertension is associated with modifiable risk factors, including an unhealthy eating habits (such as consumption of high sodium, high fat, and low vegetable diet), physical inactivity, smoking, alcohol consumption, and obesity, and non‐modifiable risk factors, such as family history, age >65 years, and comorbidities (WHO, 2021; Zhao et al., 2011). Thus, the proper and continuous management of hypertension is crucial because of its relation to various modifiable factors. However, according to the World Health Organization, 21% of adults have controlled hypertension (Mills et al., 2020; WHO, 2021). A multifactorial intervention is required to manage hypertension effectively, including treatment with antihypertensive medications, consumption of low‐calorie and low sodium diet, and performance of physical exercise (Jurik & Stastny, 2019; Unda Villafuerte et al., 2020). Particularly, adherence to antihypertensive medications is essential for reducing blood pressure levels (Muntner et al., 2022). Uncontrolled hypertension is associated with nonadherence to the medication, such as improper dosage or frequency of medication (Alsaqabi & Rabbani, 2020). Low adherence to antihypertensive medication is a noteworthy barrier in managing hypertension (Holt et al., 2010). Results of the meta‐analysis of randomized controlled trials showed that antihypertensive drugs reduced blood pressure and alleviated the risk of cardiovascular diseases (Blood Pressure Lowering Treatment Trialists Collaboration, 2021). Another meta‐analysis study reported that antihypertensive medication adherence is associated with various factors, including socioeconomic factors, such as age, civil status, education, and work status; patient‐related factors, such as health literacy and awareness, knowledge of hypertension, attitude toward hypertension, self‐efficacy, and social support; therapy‐related factors, such as drug schedule, drug usage, and alternative medicine; and condition‐related factors, such as illness perception and comorbidity (Gutierrez & Sakulbumrungsil, 2021). Antihypertensive medication adherence was associated with reduced alcohol intake and absence of co‐morbidity (Asgedom et al., 2018). Furthermore, other previous studies reported that sex is a significant factor that affects the adherence to antihypertensive drugs (Biffi et al., 2020; Chen et al., 2014).

Among various factors, health‐related quality of life (HRQoL) focuses on the impact of health status on the quality of life as a multidimensional concept, including physical, mental, and social well‐being (Yin et al., 2016). HRQoL and treatment adherence are interrelated in patient management and care (Kastien‐Hilka et al., 2017). A previous study conducted on patients with tuberculosis in South Africa reported a positive association between treatment adherence and HRQoL (Kastien‐Hilka et al., 2017). Pulmonary arterial hypertension and chronic thromboembolic pulmonary hypertension had an impact on the ability to perform daily activities (Delcroix & Howard, 2015; Ivarsson et al., 2019). In one of the results of the EuroQol 5‐Dimension Questionnaire (EQ‐5D) survey to evaluate HRQoL, patients with a low EQ‐5D index expressed more concerns about their treatment (Ivarsson et al., 2019). In particular, the management of hypertension is closely associated with HRQoL because hypertension is a chronic disease that requires consistent management. Several studies have reported that the treatments for hypertension may worsen the HRQoL (Arslantas et al., 2008; Soni et al., 2010; Xu et al., 2016). However, a low HRQoL is attributed to an individual's awareness of hypertension; thus, having a low HRQoL may also reduce treatment adherence (Xu et al., 2016). Thus, the bidirectional associations between adherence to hypertensive treatment and HRQoL should be considered, and sex differences are an important issue affecting the HRQoL. A study reported a significant sex difference in HRQoL among people with human immunodeficiency virus on antiretroviral treatment (Tesfay et al., 2015). Women with coronary artery disease showed worse HRQoL compared with men (Norris et al., 2008).

Thus, this study aimed to examine the association between nonadherence to antihypertensive medication and HRQoL. The results of this study can serve as a basis for managing HRQoL to improve patients' adherence to antihypertensive medication.

## 2 METHODS

### 2.1 Data source and participants

This study was conducted using the 5‐year data from the 2016–2020 Korea National Health and Nutrition Examination Survey (KNHANES) (KDCA, 2022). The KNHANES was provided by the Division of Health and Nutrition Survey of the Korea Disease Control and Prevention Agency (KDCA). The KNHANES is a nationally representative, population‐based, cross‐sectional survey designed to examine health‐related behaviour and health conditions. It uses a complex, multi‐stage probability sample design and examines a sample of approximately 10,000 individuals each year. This survey was collected by face to face interview or self‐report method. All survey protocols were approved by the Institutional Review Board of the KDCA. All volunteers submitted written informed consent before their participation in the study. The Institutional Review Board of Gachon University (1044396‐202206‐HR‐129‐01) waived the requirement for written consent.

**FIGURE 1:**
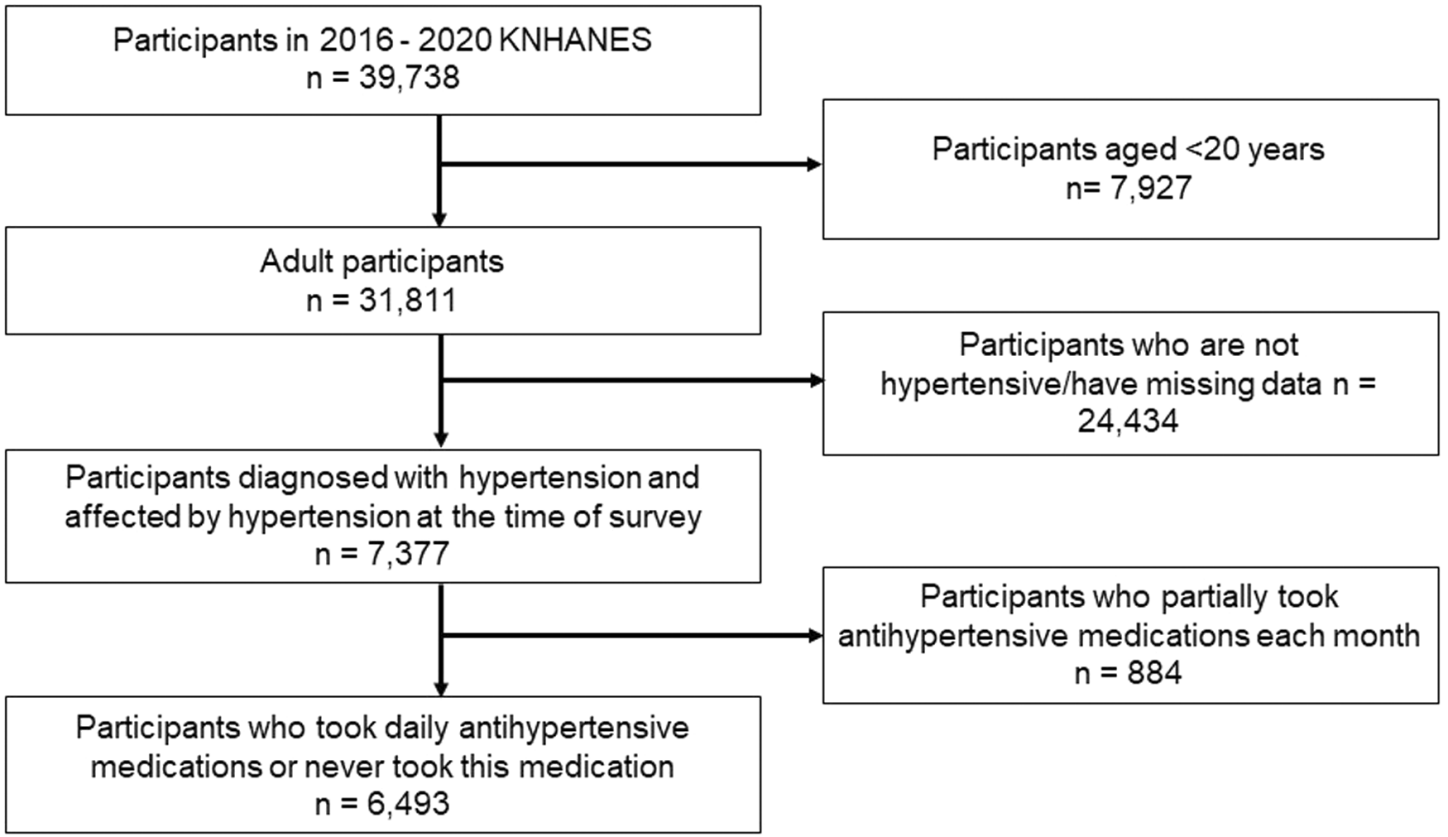
Flow chart of the study population selection.

Of the 39,738 respondents, 6493 who diagnosed with hypertension, affected by hypertension at the time of survey, and took daily antihypertensive medications or never took this medication were included in this study (Figure 1). Patients with missing data were excluded.

### 2.2 Variables

The following variables were used in this study: age, sex (male or female), education level (elementary school or below, middle school, high school, university, or above), occupation, household income (lowest, lower middle, upper middle, or highest), residential area (urban or rural), drinking (no or yes), smoking (no or yes), body mass index (BMI), cohabitation, EuroQol five‐dimensions (EQ‐5D) scores, and antihypertensive medication adherence. In terms of occupation, the participants were divided into four groups based on the major classifications of the 6th Korean Standard Classification of Occupations: white‐collar workers included managers, professionals, and office workers; pink‐collar workers included service and sales workers; blue‐collar workers included technicians and device and machine operators; agribusiness and low‐level workers included skilled workers in agriculture and fishery and low‐level laborers; and the unemployed included homemakers (Kwon et al., 2019). The BMI was classified into four groups: normal (18.5–24.9 kg/m^2^), overweight (25.0–29.9 kg/m^2^), obesity (≥30.0 kg/m^2)^, and underweight (<18.5 kg/m^2^). Cohabitation was re‐categorized as living alone and living with two or more people. The EQ‐5D was used to assess the HRQoL. Using this tool, the patients' health status was evaluated on all five dimensions: mobility (EQ1), self‐care (EQ2), usual activities (EQ3), pain/discomfort (EQ4), and anxiety/depression (EQ5). Each dimension has three response levels: no problem, some problems, and extreme problems. However, this study only analysed the following two levels: no problem and problems (including some and extreme problems). Adherence to antihypertensive medication was categorized into two groups: taking daily medication and never taking medication. Those participants who only partially took the medication each month were excluded from this study.

### 2.3 Statistical analysis

All statistical analyses were performed using the IBM SPSS Statistics version 26 (IBM). A chi‐square test or t‐test was performed to assess the differences in the participants' characteristics according to medication adherence. The categorical variables were expressed as numbers and percentages, while the continuous variables were expressed as means and standard deviations. Furthermore, logistic regression analysis was performed to determine the factors associated with nonadherence to antihypertensive medication according to sex. A *p* value of <0.05 was considered significant.

## 3 RESULTS

### 3.1 Participants' baseline characteristics according to sex

Table 1 shows the general characteristics of the 6493 hypertensive participants. Among them, 46.2% were men and 53.8% were women. Of the 2997 male participants, 87.8% responded that they lived with two or more people. Approximately 74.5% of women lived with two or more people. Of the male participants, 21.4% responded that they had problems with mobility (EQ1), 6.5% with self‐care (EQ2), 11.4% with usual activities (EQ3), 23.1% with pain/discomfort (EQ4), and 8.9% with anxiety/depression (EQ5). Of the female participants, 37.6% responded that they had problems with mobility, 10.2% with self‐care, 19.4% with usual activities, 40.0% with pain/discomfort, and 16.5% with anxiety/depression.

**TABLE 1:** Participants' baseline characteristics according to sex

Characteristics	Total (*n* = 6493)	Men (*n* = 2997)	Women (*n* = 3496)	*p*‐value
Age (years)	65.8 ± 10.9	64.1 ± 11.6	67.4 ± 10.0	<0.001
Education				
≤Elementary	2782 (42.8)	770 (25.7)	2012 (57.6)	<0.001
Middle school	1019 (15.7)	521 (17.4)	498 (14.2)	
High school	1628 (25.1)	951 (31.7)	677 (19.4)	
≥University	1064 (16.4)	755 (25.2)	309 (8.8)	
Occupation				
White collar	670 (10.3)	500 (16.7)	170 (4.9)	<0.001
Pink collar	632 (9.7)	198 (6.6)	434 (12.4)	
Blue collar	1822 (28.1)	1104 (36.8)	718 (20.5)	
Unemployed	3369 (51.9)	1195 (39.9)	2174 (62.2)	
Household income				
Lowest	2292 (35.3)	849 (28.3)	1443 (41.3)	<0.001
Lower middle	1713 (26.4)	791 (26.4)	922 (26.4)	
Upper middle	1263 (19.5)	637 (21.3)	626 (17.9)	
Highest	1225 (18.9)	720 (24.0)	505 (14.4)	
Residential area				
Urban	4843 (74.6)	2266 (75.6)	2577 (73.7)	0.080
Rural	1650 (25.4)	731 (24.4)	919 (26.3)	
Drinking				
No	2612 (40.2)	708 (23.6)	1904 (54.5)	<0.001
Yes	3881 (59.8)	2289 (76.4)	1592 (45.5)	
Smoking				
No	5558 (85.6)	2179 (72.7)	3379 (96.7)	<0.001
Yes	935 (14.4)	818 (27.3)	117 (3.3)	
BMI				
Normal	3148 (48.5)	1408 (47.0)	1740 (49.8)	<0.001
Overweight	2700 (41.6)	1335 (44.5)	1365 (39.0)	
Obesity	564 (8.7)	213 (7.1)	351 (10.0)	
Underweight	81 (1.2)	41 (1.4)	40 (1.1)	
Cohabitation				
Alone	1260 (19.4)	367 (12.2)	893 (25.5)	<0.001
Living together (≥2)	5233 (80.6)	2630 (87.8)	2603 (74.5)	
Mobility (EQ1)				
No problem	4540 (69.9)	2357 (78.6)	2183 (62.4)	<0.001
Problem	1953 (30.1)	640 (21.4)	1313 (37.6)	
Self‐care (EQ2)				
No problem	5942 (91.5)	2801 (93.5)	3141 (89.8)	<0.001
Problem	551 (8.5)	196 (6.5)	355 (10.2)	
Usual activities (EQ3)				
No problem	5473 (84.3)	2654 (88.6)	2819 (80.6)	<0.001
Problem	1020 (15.7)	343 (11.4)	677 (19.4)	
Pain/discomfort (EQ4)				
No problem	4402 (67.8)	2306 (76.9)	2096 (60.0)	<0.001
Problem	2091 (32.2)	691 (23.1)	1400 (40.0)	
Anxiety/depression (EQ5)				
No problem	5649 (87.0)	2729 (91.1)	2920 (83.5)	<0.001
Problem	844 (13.0)	268 (8.9)	576 (16.5)	
Medication				
Everyday	6262 (96.4)	2848 (95.0)	3414 (97.7)	<0.001
None	231 (3.6)	149 (5.0)	82 (2.3)	

*Note*: Values are presented as the mean ± standard deviation or *n* (%).

Abbreviation: EQ, EuroQol.

### 3.2 Characteristics of the participants according to antihypertensive medication adherence

Table 2 presents the characteristics of the participants according to adherence to antihypertensive medication in men and women. In men, antihypertensive medication nonadherence showed significant differences in terms of age, education level, occupation, smoking, cohabitation, and anxiety/depression (EQ5). In women, antihypertensive medication nonadherence showed significant differences in terms of age, education, occupation, and mobility (EQ1).

**TABLE 2:** Participants' characteristics according to antihypertensive medication nonadherence

Characteristics	Men			Women		
	Every day (*n* = 2848)	None (*n* = 149)	*p*‐value	Every day (*n* = 3414)	None (*n* = 82)	*p*‐value
Age (years)	64.6 ± 11.0	54.0 ± 16.0	**<0.001**	67.5 ± 9.9	60.7 ± 11.7	**<0.001**
Education						
≤Elementary	743 (26.1)	27 (18.1)	**0.009**	1979 (58.0)	33 (40.2)	**0.001**
Middle school	502 (17.6)	19 (12.8)		485 (14.2)	13 (15.9)	
High school	900 (31.6)	51 (34.2)		657 (19.2)	20 (24.4)	
≥University	703 (24.7)	52 (34.9)		293 (8.6)	16 (19.5)	
Occupation						
White collar	463 (16.3)	37 (24.8)	**0.014**	160 (4.7)	10 (12.2)	**<0.001**
Pink collar	184 (6.5)	14 (9.4)		415 (12.2)	19 (23.2)	
Blue collar	1058 (37.1)	46 (30.9)		705 (20.7)	13 (15.9)	
Unemployed	1143 (40.1)	52 (34.9)		2134 (62.5)	40 (48.8)	
Household income						
Lowest	807 (28.3)	42 (28.2)	0.491	1415 (41.4)	28 (34.1)	0.325
Lower middle	759 (26.7)	32 (21.5)		901 (26.4)	21 (25.6)	
Upper middle	603 (21.2)	34 (22.8)		610 (17.9)	16 (19.5)	
Highest	679 (23.8)	41 (27.5)		488 (14.3)	17 (20.7)	
Residential area						
Urban	2149 (75.5)	117 (78.5)	0.395	2512 (73.6)	65 (79.3)	0.247
Rural	699 (24.5)	32 (21.5)		902 (26.4)	17 (20.7)	
Drinking						
No	679 (23.8)	29 (19.5)	0.220	1866 (54.7)	38 (46.3)	0.135
Yes	2169 (76.2)	120 (80.5)		1548 (45.3)	44 (53.7)	
Smoking						
No	2087 (73.3)	92 (61.7)	**0.002**	3300 (96.7)	79 (96.3)	0.874
Yes	761 (26.7)	57 (38.3)		114 (3.3)	3 (3.7)	
BMI						
Normal	1340 (47.1)	68 (45.6)	0.449	1689 (49.5)	51 (62.2)	0.052
Overweight	1272 (44.7)	63 (42.3)		1344 (39.4)	21 (25.6)	
Obesity	198 (7.0)	15 (10.1)		343 (10.0)	8 (9.8)	
Underweight	38 (1.3)	3 (2.0)		38 (1.1)	2 (2.4)	
Cohabitation						
Alone	339 (11.9)	28 (18.8)	**0.012**	872 (25.5)	21 (25.6)	0.989
Living together (≥2)	2509 (88.1)	121 (81.2)		2542 (74.5)	61 (74.4)	
Mobility (EQ1)						
No problem	2239 (78.6)	118 (79.2)	0.867	2118 (62.0)	65 (79.3)	**0.001**
Problem	609 (21.4)	31 (20.8)		1296 (38.0)	17 (20.7)	
Self‐care (EQ2)						
No problem	2663 (93.5)	138 (92.6)	0.670	3064 (89.7)	77 (93.9)	0.218
Problem	185 (6.5)	11 (7.4)		350 (10.3)	5 (6.1)	
Usual activities (EQ3)						
No problem	2526 (88.7)	128 (85.9)	0.297	2750 (80.6)	69 (84.1)	0.415
Problem	322 (11.3)	21 (14.1)		664 (19.4)	13 (15.9)	
Pain/discomfort (EQ4)						
No problem	2198 (77.2)	108 (72.5)	0.185	2047 (60.0)	49 (59.8)	0.970
Problem	650 (22.8)	41 (27.5)		1367 (40.0)	33 (40.2)	
Anxiety/depression (EQ5)						
No problem	2604 (91.4)	125 (83.9)	**0.002**	2857 (83.7)	63 (76.8)	0.098
Problem	244 (8.6)	24 (16.1)		557 (16.3)	19 (23.2)	

*Note*: Values are presented as the mean ± standard deviation or *n* (%).

Abbreviation: EQ, EuroQol.

Bold values indicate significant (*p* < 0.05).

### 3.3 Factors associated with antihypertensive medication nonadherence according to sex

The results of the multiple logistic regression analyses between antihypertensive medication nonadherence and cohabitation or EQ‐5D are presented in Table 3. After adjusting for age, education, occupation, and smoking, the odds ratio (OR) for antihypertensive medication nonadherence in men was 0.59 (95% confidence interval (CI), 0.37–0.94) for those living together. The OR for antihypertensive medication nonadherence due to anxiety/depression (EQ5) was 1.82 (95% CI, 1.04–3.17). In women, the OR values for antihypertensive medication nonadherence due to problems with mobility (EQ1) and anxiety/depression (EQ5) were 0.39 (95% CI, 0.19–0.81) and 1.96 (95% CI, 1.09–3.54), respectively.

**TABLE 3:** Factors associated with antihypertensive medication nonadherence according to sex

Characteristics	Men		Women	
	Crude OR (CI)	Adjusted	Crude	Adjusted
Living together (ref = alone)	**0.63 (0.41–0.63)** *	**0.59 (0.37–0.94)** *	0.91 (0.55–1.52)	0.61 (0.36–1.05)
Mobility (EQ1) (ref = no)	0.68 (0.39–1.18)	1.15 (0.63–2.07)	**0.29 (0.15–0.58)** ***	**0.39 (0.19–0.81)** *
Self‐care (EQ2) (ref = no)	0.91 (0.42–1.97)	1.03 (0.45–2.34)	0.60 (0.21–1.69)	0.72 (0.25–2.05)
Usual activities (EQ3) (ref = no)	1.18 (0.59–2.35)	1.11 (0.53–2.31)	1.31 (0.59–2.91)	1.46 (0.64–3.34)
Pain/discomfort (EQ4) (ref = no)	1.18 (0.75–1.86)	1.17 (0.72–1.91)	1.38 (0.81–2.35)	1.42 (0.83–2.45)
Anxiety/depression (EQ5) (ref = no)	**1.95 (1.15–3.30)** *	**1.82 (1.04–3.17)** *	**2.03 (1.14–3.61)** *	**1.96 (1.09–3.54)** *

*Note*: Adjusted for age, education, occupation, and smoking.

Abbreviations: CI, confidence interval; EQ, EuroQol; OR, odds ratio; ref, reference group.

* *p* < 0.05.

*** *p* < 0.001.

Bold values indicate significant (*p* < 0.05).

## 4 DISCUSSION

In this study, we focused on determining the factors related to antihypertensive medication nonadherence according to sex. Our results showed that anxiety/depression was positively associated with antihypertensive medication nonadherence, regardless of sex. According to sex, mobility was negatively associated with antihypertensive medication nonadherence in women, but not in men. In men, living together was negatively related to antihypertensive medication nonadherence. These results may serve as a basis for selecting the appropriate nurse‐based interventions to improve medication adherence.

Health‐related quality of life is a multidimensional concept related to physical, psychological, and social functioning that is affected by disease and/or treatment (Megari, 2013). In general, chronic diseases negatively affect the quality of life as long‐term treatment and management continue. Hypertension, which is one of the common chronic diseases, is closely associated with the HRQoL (Soni et al., 2010). Hypertension awareness was associated with lower HRQOL in hypertensive patients (Mi et al., 2015). On the contrary, hypertensive patients with poor HRQoL had more concerns about treatment and lower coping ability (Ivarsson et al., 2019). Lower HRQoL affects medication adherence, which is important for managing hypertension (Holt et al., 2010). Overall, poor HRQoL is related to severe hypertension (Alsaqabi & Rabbani, 2020). The bidirectional associations between hypertension and HRQoL may lead to a vicious cycle. Thus, the HRQoL is important for improving adherence to antihypertensive medication.

EuroQol 5‐Dimension Questionnaire, which is used to assess HRQoL, comprises five dimensions: mobility, self‐care, usual activities, pain/discomfort, and anxiety/depression. In this study, poor adherence to antihypertensive medication was associated with increased levels of anxiety and depression. Nonadherence to medication is a major issue in the treatment of major depressive disorders (Ho et al., 2017). A previous qualitative study reported that the facilitators of antidepressant adherence were insight, perceived health benefits, and regular activities (Ho et al., 2017). These depressive symptoms affect the antihypertensive medication adherence of all participants as they affect cognitive insight and regular activities (Minaeva et al., 2020; Palmer et al., 2015). In this study, poor mobility significantly lowered antihypertensive medication nonadherence in women. Views on sex‐based differences in medication adherence vary, but this remains an important issue (Chen et al., 2014; Kitaoka et al., 2016; Mahmoodi et al., 2019; Soni et al., 2010; Xu et al., 2016). No sex difference was observed in the medication adherence among adolescent kidney transplant recipients, but women showed better medication adherence than men among young adults (Boucquemont et al., 2019). A previous study reported that women with hypertension were more at risk of poor HRQoL than men (Xiao et al., 2019). Another study reported that women with hypertension had lower general health perceptions, while hypertension was related to higher social functioning in men (Kitaoka et al., 2016). In this study, explaining the relationship between poor mobility in HRQoL and low antihypertensive medication nonadherence in women remains challenging. However, psychological factors, such as anxiety and depression, act as risk factors for increased hypertensive medication nonadherence, while physical factors, such as poor mobility, act as warning signs to lower hypertensive medication nonadherence.

Another interesting finding is that living together was related to lower antihypertensive medication nonadherence among men. Medication adherence is closely associated with social support, such as family and friends, in patients with hypertension (Scheurer et al., 2012; Shahin et al., 2021). Another study reported that social support was related to adherence to depression treatment according to gender and race (Gerlach et al., 2017). This study showed that men with hypertension were more susceptible to receiving social support than women with antihypertensive medication nonadherence.

### 4.1 Implication

In particular, nurse‐based interventions are important to improve medication adherence and manage the HRQoL (Chow & Wong, 2010; Dijkstra et al., 2021). The medication adherence support intervention provided by home care nurses increased satisfaction and improved the self‐management of medication (Dijkstra et al., 2021). The nurse‐based case management program was effective in improving the HRQoL of peritoneal dialysis patients (Chow & Wong, 2010). Thus, the results of this study may serve as a basis for determining the appropriate nursing interventions by understanding the factors associated with antihypertensive medication adherence.

### 4.2 Limitation

However, our cross‐sectional study has limitations in elucidating the association between HRQoL and adherence to antihypertensive medication. It is difficult to establish a cause and effect relationship. Thus, further studies are warranted to demonstrate the causal relationship between HRQoL and adherence to antihypertensive medications. Furthermore, the variables were limited to questionnaires based on Korea National Health and Nutrition Examination Survey. Sufficient variables related to antihypertensive medication nonadherence such as comorbidities were not considered in this study because variables appropriate for the purpose of this study were selected and analysed based on limited questionnaires. It is necessary to conduct further studies in consideration of the various factors related to antihypertensive medication nonadherence.

## 5 CONCLUSION

This study showed the factors associated with antihypertensive medication nonadherence according to sex. HRQoL was associated with antihypertensive medication nonadherence. Understanding the factors associated with medication adherence may help determine the appropriate nursing intervention. Our results can serve as a basis for managing the health‐related quality of life to improve adherence to antihypertensive medication.

## FUNDING INFORMATION

This research has been supported by the AMOREPACIFIC Foundation.

## CONFLICT OF INTEREST

The authors declare that they have no competing interest.

## ETHICAL APPROVAL

It was provided by the Division of Health and Nutrition Survey of the KDCA. All survey protocols were approved by the Institutional Review Board of the KDCA. All volunteers submitted written informed consent prior to their participation in the study. The Institutional Review Board of Gachon University (1044396‐202,206‐HR‐129‐01) waived the requirement for written consent.

## Data Availability

All data used were from the 2016–2020 Korea National Health and Nutrition Examination Survey conducted by the Division of Health and Nutrition Survey of the Korea Disease Control and Prevention Agency.

## References

[nop21599-bib-0001] Alsaqabi, Y. S. , & Rabbani, U. (2020). Medication adherence and its association with quality of life among hypertensive patients attending primary health care centers in Saudi Arabia. Cureus, 12(12), e11853. 10.7759/cureus.11853 33282607 PMC7714734

[nop21599-bib-0002] Arslantas, D. , Ayranci, U. , Unsal, A. , & Tozun, M. (2008). Prevalence of hypertension among individuals aged 50 years and over and its impact on health related quality of life in a semi‐rural area of western Turkey. Chinese Medical Journal, 121(16), 1524–1531. 18982863

[nop21599-bib-0003] Asgedom, S. W. , Atey, T. M. , & Desse, T. A. (2018). Antihypertensive medication adherence and associated factors among adult hypertensive patients at Jimma University specialized hospital, Southwest Ethiopia. BMC Research Notes, 11(1), 27. 10.1186/s13104-018-3139-6 29335003 PMC5769214

[nop21599-bib-0004] Biffi, A. , Rea, F. , Iannaccone, T. , Filippelli, A. , Mancia, G. , & Corrao, G. (2020). Sex differences in the adherence of antihypertensive drugs: A systematic review with meta‐analyses. BMJ Open, 10(7), e036418. 10.1136/bmjopen-2019-036418 PMC7348648 32641331

[nop21599-bib-0005] Blood Pressure Lowering Treatment Trialists Collaboration . (2021). Pharmacological blood pressure lowering for primary and secondary prevention of cardiovascular disease across different levels of blood pressure: An individual participant‐level data meta‐analysis. Lancet, 397(10285), 1625–1636. 10.1016/S0140-6736(21)00590-0 33933205 PMC8102467

[nop21599-bib-0006] Boucquemont, J. , Pai, A. L. H. , Dharnidharka, V. R. , Hebert, D. , Furth, S. L. , & Foster, B. J. (2019). Gender differences in medication adherence among adolescent and young adult kidney transplant recipients. Transplantation, 103(4), 798–806. 10.1097/TP.0000000000002359 29994983 PMC6326890

[nop21599-bib-0007] CDC . (2021). Facts about hypertension. Centers for Disease Control and Prevention.

[nop21599-bib-0008] Chen, S. L. , Lee, W. L. , Liang, T. , & Liao, I. C. (2014). Factors associated with gender differences in medication adherence: A longitudinal study. Journal of Advanced Nursing, 70(9), 2031–2040. 10.1111/jan.12361 24506542

[nop21599-bib-0009] Chow, S. K. , & Wong, F. K. (2010). Health‐related quality of life in patients undergoing peritoneal dialysis: Effects of a nurse‐led case management programme. Journal of Advanced Nursing, 66(8), 1780–1792. 10.1111/j.1365-2648.2010.05324.x 20557392

[nop21599-bib-0010] Delcroix, M. , & Howard, L. (2015). Pulmonary arterial hypertension: The burden of disease and impact on quality of life. European Respiratory Review, 24(138), 621–629. 10.1183/16000617.0063-2015 26621976 PMC9487616

[nop21599-bib-0011] Dijkstra, N. E. , Vervloet, M. , Sino, C. G. M. , Heerdink, E. R. , Nelissen‐Vrancken, M. , Bleijenberg, N. , de Bruin, M. , & Schoonhoven, L. (2021). Home care patients' experiences with home care nurses' support in medication adherence. Patient Preference and Adherence, 15, 1929–1940. 10.2147/PPA.S302818 34511888 PMC8420798

[nop21599-bib-0012] Gerlach, L. B. , Kavanagh, J. , Watkins, D. , Chiang, C. , Kim, H. M. , & Kales, H. C. (2017). With a little help from my friends?: Racial and gender differences in the role of social support in later‐life depression medication adherence. International Psychogeriatrics, 29(9), 1485–1493. 10.1017/S104161021700076X 28528594

[nop21599-bib-0013] Gutierrez, M. M. , & Sakulbumrungsil, R. (2021). Factors associated with medication adherence of hypertensive patients in The Philippines: A systematic review. Clinical Hypertension, 27(1), 19. 10.1186/s40885-021-00176-0 34593047 PMC8485436

[nop21599-bib-0014] Ho, S. C. , Jacob, S. A. , & Tangiisuran, B. (2017). Barriers and facilitators of adherence to antidepressants among outpatients with major depressive disorder: A qualitative study. PLoS ONE, 12(6), e0179290. 10.1371/journal.pone.0179290 28614368 PMC5470687

[nop21599-bib-0015] Holt, E. W. , Muntner, P. , Joyce, C. J. , Webber, L. , & Krousel‐Wood, M. A. (2010). Health‐related quality of life and antihypertensive medication adherence among older adults. Age and Ageing, 39(4), 481–487. 10.1093/ageing/afq040 20513770 PMC2886202

[nop21599-bib-0016] Ivarsson, B. , Hesselstrand, R. , Radegran, G. , & Kjellstrom, B. (2019). Health‐related quality of life, treatment adherence and psychosocial support in patients with pulmonary arterial hypertension or chronic thromboembolic pulmonary hypertension. Chronic Respiratory Disease, 16, 1479972318787906. 10.1177/1479972318787906 30011997 PMC6302968

[nop21599-bib-0017] Jurik, R. , & Stastny, P. (2019). Role of nutrition and exercise programs in reducing blood pressure: A systematic review. Journal of Clinical Medicine, 8(9), 1393. 10.3390/jcm8091393 31492032 PMC6780911

[nop21599-bib-0018] Kastien‐Hilka, T. , Rosenkranz, B. , Schwenkglenks, M. , Bennett, B. M. , & Sinanovic, E. (2017). Association between health‐related quality of life and medication adherence in pulmonary tuberculosis in South Africa. Frontiers in Pharmacology, 8, 919. 10.3389/fphar.2017.00919 29326591 PMC5741974

[nop21599-bib-0019] KDCA . (2022). Korea national health & nutrition examination survey. Korea Disease Control and Prevention Agency.

[nop21599-bib-0020] Kitaoka, M. , Mitoma, J. , Asakura, H. , Anyenda, O. E. , Nguyen, T. T. , Hamagishi, T. , Hori, D. , Suzuki, F. , Shibata, A. , Horii, M. , Tsujiguchi, H. , Hibino, Y. , Kambayashi, Y. , Hitomi, Y. , Shikura, N. , & Hiroyuki, N. (2016). The relationship between hypertension and health‐related quality of life: Adjusted by chronic pain, chronic diseases, and life habits in the general middle‐aged population in Japan. Environmental Health and Preventive Medicine, 21(4), 193–214. 10.1007/s12199-016-0514-6 26893020 PMC4907927

[nop21599-bib-0021] Kwon, S. , Kim, W. , Yang, S. , & Choi, K. H. (2019). Influence of the type of occupation on osteoarthritis of the knee in men: The Korean National Health and nutrition examination survey 2010‐2012. Journal of Occupational Health, 61(1), 54–62. 10.1002/1348-9585.12022 30698336 PMC6499360

[nop21599-bib-0022] Mahmoodi, H. , Jalalizad Nahand, F. , Shaghaghi, A. , Shooshtari, S. , Jafarabadi, M. A. , & Allahverdipour, H. (2019). Gender based cognitive determinants of medication adherence In older adults with chronic conditions. Patient Preference and Adherence, 13, 1733–1744. 10.2147/PPA.S219193 31686791 PMC6800551

[nop21599-bib-0023] Megari, K. (2013). Quality of life in chronic disease patients. Health Psychology Research, 1(3), e27. 10.4081/hpr.2013.e27 26973912 PMC4768563

[nop21599-bib-0024] Mi, B. , Dang, S. , Li, Q. , Zhao, Y. , Yang, R. , Wang, D. , & Yan, H. (2015). Association between awareness of hypertension and health‐related quality of life in a cross‐sectional population‐based study in rural area of Northwest China. Medicine (Baltimore), 94(29), e1206. 10.1097/MD.0000000000001206 26200639 PMC4603002

[nop21599-bib-0025] Mills, K. T. , Stefanescu, A. , & He, J. (2020). The global epidemiology of hypertension. Nature Reviews. Nephrology, 16(4), 223–237. 10.1038/s41581-019-0244-2 32024986 PMC7998524

[nop21599-bib-0026] Minaeva, O. , Booij, S. H. , Lamers, F. , Antypa, N. , Schoevers, R. A. , Wichers, M. , & Riese, H. (2020). Level and timing of physical activity during normal daily life in depressed and non‐depressed individuals. Translational Psychiatry, 10(1), 259. 10.1038/s41398-020-00952-w 32732880 PMC7393081

[nop21599-bib-0027] Muntner, P. , Miles, M. A. , Jaeger, B. C. , Hannon, L., 3rd , Hardy, S. T. , Ostchega, Y. , Wozniak, G. , & Schwartz, J. E. (2022). Blood pressure control among US adults, 2009 to 2012 through 2017 to 2020. Hypertension, 79, 1971–1980. 10.1161/HYPERTENSIONAHA.122.19222 35616029 PMC9370255

[nop21599-bib-0028] Norris, C. M. , Spertus, J. A. , Jensen, L. , Johnson, J. , Hegadoren, K. M. , Ghali, W. A. , & Investigators, A. (2008). Sex and gender discrepancies in health‐related quality of life outcomes among patients with established coronary artery disease. Circulation. Cardiovascular Quality and Outcomes, 1(2), 123–130. 10.1161/CIRCOUTCOMES.108.793448 20031799

[nop21599-bib-0029] Palmer, E. C. , Gilleen, J. , & David, A. S. (2015). The relationship between cognitive insight and depression in psychosis and schizophrenia: A review and meta‐analysis. Schizophrenia Research, 166(1–3), 261–268. 10.1016/j.schres.2015.05.032 26095015

[nop21599-bib-0030] Scheurer, D. , Choudhry, N. , Swanton, K. A. , Matlin, O. , & Shrank, W. (2012). Association between different types of social support and medication adherence. The American Journal of Managed Care, 18(12), e461–e467. 23286676

[nop21599-bib-0031] Shahin, W. , Kennedy, G. A. , & Stupans, I. (2021). The association between social support and medication adherence in patients with hypertension: A systematic review. Pharmacy Practice (Granada), 19(2), 2300. 10.18549/PharmPract.2021.2.2300 PMC8234709 34221197

[nop21599-bib-0032] Soni, R. K. , Porter, A. C. , Lash, J. P. , & Unruh, M. L. (2010). Health‐related quality of life in hypertension, chronic kidney disease, and coexistent chronic health conditions. Advances in Chronic Kidney Disease, 17(4), e17–e26. 10.1053/j.ackd.2010.04.002 20610351 PMC2901238

[nop21599-bib-0033] Tesfay, A. , Gebremariam, A. , Gerbaba, M. , & Abrha, H. (2015). Gender differences in health related quality of life among people living with HIV on highly active antiretroviral therapy in Mekelle town, Northern Ethiopia. Biomed Research International, 2015, 516369. 10.1155/2015/516369 25632393 PMC4303010

[nop21599-bib-0034] Unda Villafuerte, F. , Llobera Canaves, J. , Lorente Montalvo, P. , Moreno Sancho, M. L. , Oliver Oliver, B. , Bassante Flores, P. , Estela Mantolan, A. , Pou Bordoy, J. , Rodríguez Ruiz, T. , Requena Hernández, A. , Leiva, A. , Torrent Quetglas, M. , Coll Benejam, J. M. , D'Agosto Forteza, P. , Rigo Carratalà, F. , & The Medichy Group . (2020). Effectiveness of a multifactorial intervention, consisting of self‐management of antihypertensive medication, self‐measurement of blood pressure, hypocaloric and low sodium diet, and physical exercise, in patients with uncontrolled hypertension taking 2 or more antihypertensive drugs: The MEDICHY study. Medicine (Baltimore), 99(17), e19769. 10.1097/MD.0000000000019769 32332617 PMC7220514

[nop21599-bib-0035] WHO . (2021). Hypertension. World Health Organization.

[nop21599-bib-0036] Xiao, M. , Zhang, F. , Xiao, N. , Bu, X. , Tang, X. , & Long, Q. (2019). Health‐related quality of life of hypertension patients: A population‐based cross‐sectional study in Chongqing, China. International Journal of Environmental Research and Public Health, 16(13), 2348. 10.3390/ijerph16132348 31277210 PMC6652141

[nop21599-bib-0037] Xu, X. , Rao, Y. , Shi, Z. , Liu, L. , Chen, C. , & Zhao, Y. (2016). Hypertension impact on health‐related quality of life: A cross‐sectional survey among middle‐aged adults in Chongqing, China. International Journal of Hypertension, 2016, 7404957. 10.1155/2016/7404957 27630771 PMC5005589

[nop21599-bib-0038] Yin, S. , Njai, R. , Barker, L. , Siegel, P. Z. , & Liao, Y. (2016). Summarizing health‐related quality of life (HRQOL): Development and testing of a one‐factor model. Population Health Metrics, 14, 22. 10.1186/s12963-016-0091-3 27408606 PMC4940947

[nop21599-bib-0039] Zhao, D. , Qi, Y. , Zheng, Z. , Wang, Y. , Zhang, X. Y. , Li, H. J. , Liu, H. H. , Zhang, X. T. , Du, J. , & Liu, J. (2011). Dietary factors associated with hypertension. Nature Reviews. Cardiology, 8(8), 456–465. 10.1038/nrcardio.2011.75 21727918

